# The feasibility, patterns of use and acceptability of using mobile phone text-messaging to improve treatment adherence and post-treatment review of children with uncomplicated malaria in western Kenya

**DOI:** 10.1186/1475-2875-13-44

**Published:** 2014-02-03

**Authors:** Gabriel Otieno, Sophie Githinji, Caroline Jones, Robert W Snow, Ambrose Talisuna, Dejan Zurovac

**Affiliations:** 1 Malaria Public Health Department, KEMRI-Wellcome Trust-University of Oxford Research Programme, Nairobi, Kenya; 2 Centre for Tropical Medicine, Nuffield Department of Clinical Medicine, University of Oxford, Oxford, UK; 3 Center for Global Health and Development, Boston University School of Public Health, Boston, Massachusetts, USA

## Abstract

**Background:**

Trials evaluating the impact of mobile phone text-messaging to support management of acute diseases, such as malaria, are urgently needed in Africa. There has been however a concern about the feasibility of interventions that rely on access to mobile phones among caregivers in rural areas. To assess the feasibility and inform development of an intervention to improve adherence to malaria medications and post-treatment review, mobile phone network, access, ownership and use among caregivers in western Kenya was assessed.

**Methods:**

A cross-sectional survey based on outpatient exit interviews was undertaken among caregivers of children with malaria at four trial facilities. The main outcomes were proportions of caregivers that have mobile signal at home; have access to mobile phones; are able to read; and use text-messaging. Willingness to receive text-message reminders was also explored. Descriptive analyses were performed.

**Results:**

Of 400 interviewed caregivers, the majority were female (93.5%), mothers of the sick children (87.8%) and able to read (97.3%). Only 1.7% of caregivers were without any education. Nearly all (99.8%) reported access to a mobile signal at home. 93.0% (site range: 89-98%) had access to a mobile phone within their household while 73.8% (site range: 66-78%) possessed a personal phone. Among caregivers with mobile phone access, 93.6% (site range: 85-99%) used the phone to receive text-messages. Despite only 19% having electricity at home nearly all (99.7%) caregivers reported that they would be able to have permanent phone access to receive text-messages in the next 28 days. Willingness to receive text-message reminders was nearly universal (99.7%) with 41.7% of caregivers preferring texts in English, 32.3% in Kiswahili and 26.1% in Dholuo.

**Conclusions:**

Despite concerns that the feasibility of text-messaging interventions targeting caregivers may be compromised in rural high malaria risk areas in Kenya, very favourable conditions were found with respect to mobile network, access and ownership of phones, use of text-messaging and minimum literacy levels required for successful intervention delivery. Moreover, there was a high willingness of caregivers to receive text-message reminders. Impact evaluations of carefully tailored text-messaging interventions targeting caregivers of children with malaria are timely and justified.

## Background

The expansion of mobile network coverage, the rapid growth in mobile phone penetration and decreasing costs of phone services has been seen as an opportunity to overcome communication, infrastructure and human resource weaknesses of health systems in Africa [1,2]. This could potentially result in improved medical and public health practice - the relatively new concept known as mobile health or mHealth [2]. Despite limited impact evidence of mHealth in Africa [3-6], several patient targeted trials based on the least expensive text-messaging phone function have shown encouraging results in improving adherence to medications [7,8], post-operative clinic visits [9], and skilled delivery attendance [10]. Interestingly, while most studies focused on chronic conditions and long-term therapies, the trials evaluating impact of text-messaging to support management of acute diseases have rarely been performed [11]. This could be of particular interest for malaria, a major cause of death in young children in Africa whose management is commonly characterized by patients’ non-adherence to complex dosing regimens, weak follow up and high risks of poor clinical outcomes and anti-malarial drug resistance [12-17].

Despite growing trends in network and mobile phone coverage across Africa, it has been found that even in the countries with high penetration such as Kenya, the basic technology interventions using low-end mobile phones and text-messaging may be subject to access disparities with respect to geographical area, gender, age, education, literacy, urbanization and poverty [18,19]. Such disparities are of concern for malaria control where populations at highest risk of poor outcomes are rural communities with coincidental lower levels of literacy and socioeconomic status.

A multi-centre trial is currently prepared at Kenyan public health facilities to evaluate the effects of text-message reminders on adherence to medications and post-treatment review for children with uncomplicated malaria. The intervention conceptually entails automated one-way distribution of text-message reminders timed to the dosing of the first-line therapy for malaria in Kenya, artemether-lumefantrine (AL). The text-messages will follow the recommended six-dose AL regimen over three days with follow-up reminders to bring children back to the facility for clinical and parasitological evaluation on day 4, day 28 and any time in-between if the child does not get better. To assess feasibility and inform development of the intervention, mobile phone access, ownership and use among caregivers attending the trial sites was assessed and their willingness to receive the intervention was explored.

## Methods

### Description of study area

**Figure 1:**
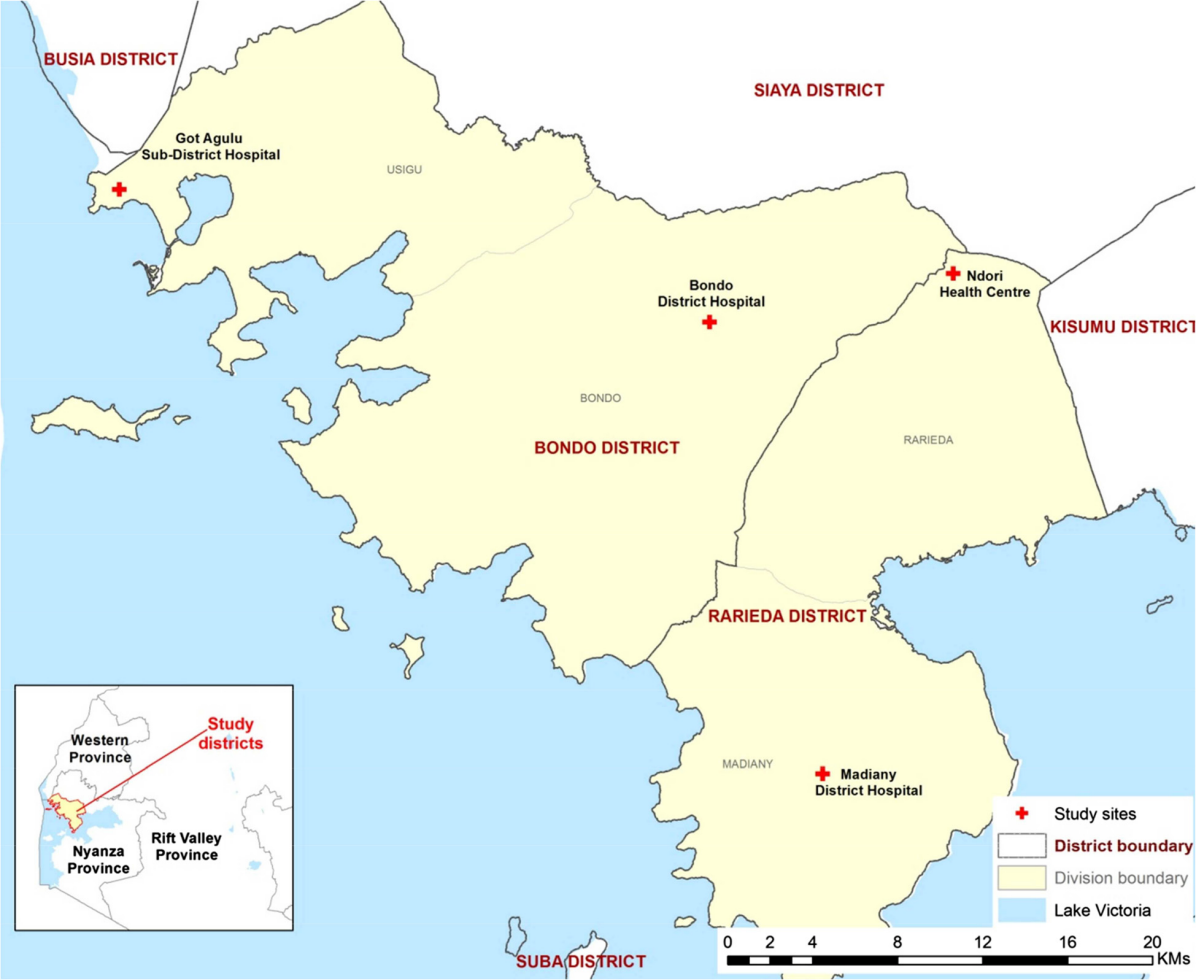
The map of Bondo and Rarieda districts with study health facilities.

The study sites include four public health facilities in Bondo and Rarieda districts of Siaya County in Kenya (Figure 1). According to the latest 2009 census, Bondo and Rarieda districts have a combined population of 292,080 with a density of 997 persons per km^2^. The population is predominantly of Luo ethnicity and earns its living through subsistence farming and fishing. The area is largely rural with urban settlement limited to one town, Bondo. Bondo and Rarieda districts are located in Nyanza Province, which has the highest infant and under-five mortality rates in Kenya of 95 and 149 deaths per 1,000 live births respectively [20]. In 2008, the respective infant and under-five mortality rates of 105 and 212 deaths/1,000 live births were reported within the Health and Demographic Surveillance System area, which covers part of Rarieda district [21]. Malaria transmission is high with seasonal peaks in May–July and October–November. In 2010, *Plasmodium falciparum* prevalence rates in children aged 2–10 years (PfPR_2-10_) were estimated at 49.4% across Siaya County with 41.5% of the population living in areas where PfPR_2-10_ is greater than 50% [22]. Of 52 public health facilities in Bondo and Rarieda districts, 14 have a laboratory capacity for malaria microscopy of which the following four were selected for the trial sites: Bondo and Madiany district hospitals, Got-Agulu sub-district hospital and Ndori health centre. Bondo District Hospital is the referral facility and the only one located in urban area.

### Data collection and definitions

A cross-sectional quantitative survey based on outpatient exit interviews was undertaken at each of four study facilities until 100 consecutive participants were enrolled at each site. Data were collected between 2nd and 30th July 2013. All caregivers of sick children underwent rapid screening when they were ready to leave the facility. The inclusion criteria for the exit interview reflected those for the trial and included: caregivers of children below five years of age; those with children treated for malaria on an outpatient basis without being referred for hospitalization; and those caregivers who would be administering the 3-day course of malaria medications at home. At each site data were collected by a trained study nurse experienced in exit interviews and prior mHealth assessments [19]. During the interviews, caregivers were asked about their demographics; education level and literacy; availability of mobile phone networks at home; patterns of access, ownership and use of phones; willingness to receive text-message reminders on administering malaria medications and when to come back to the facility; and language preferences for messages. Finally, the brand and model of mobile phones was recorded for all respondents who brought the phone to the health facility on the survey day.

### Data management and statistical analysis

All paper-based questionnaires were entered daily in Access data base (Microsoft, USA) by the data manager who was also the supervisor of data collection. All analyses were performed using STATA, version 11 (Stata Corp, College Station, Texas). Descriptive analyses reporting frequencies were undertaken for each study facility and combined for the four sites. The primary study outcomes determining feasibility of the text-messaging intervention were proportions of caregivers: 1) residing at a home with a mobile phone network; 2) having access to mobile phones; 3) demonstrating basic literacy levels; and 4) using text-messaging mobile phone function. Caregiver was defined as a person bringing a sick child to the health facility and administering malaria medicines at home. Education levels were defined as the highest level of education reached or completed. Availability of a mobile phone network at home was determined using a proxy measures based on the caregiver’s report. Access to a mobile phone was defined as either a personally owned phone or access to someone else’s phone within the same household. To reflect the one-way distribution of the intervention, the use of text-messaging was defined as receiving texts among caregivers with access to mobile phones. Similarly, caregiver’s literacy was defined as their ability to read three main languages (English, Kiswahili and Dholuo) tested by interviewers. In addition to text-messaging, the use of mobile phones was assessed for voice communication, mobile money transfers, internet browsing, and e-mail communication among other phone functions. For those caregivers who brought a phone to the facility, their devices were classified as basic (voice and text-messaging only), medium level (limited data transfer possible but not on “smartphone” operating systems) and “smartphones” (devices with Android, Symbian, iOS and Blackberry operating systems).

### Ethical approval

All caregivers provided written informed consent prior to interviews. The study protocol was approved by the University of Oxford (OXTREC-1011-13) and the Kenya Medical Research Institute (SSC No 2554).

## Results

### General characteristics of caregivers

Of 819 caregivers attending outpatient consultation with a sick child, the most common reason for study exclusion was absence of malaria treatment (371; 45.3%) followed by child’s age (child older than 5 years) (66; 8.1%), administration of medicines at home by another person (27; 3.3%) and child’s referral for hospitalization (15; 1.8%). Of 400 caregivers meeting the inclusion criteria, none of them refused consent and all completed interviews. The characteristics of caregivers were similar across all sites (Table 1). The large majority were female (93.5%), mothers of the sick children (87.8%) and with an average age of 27 years. Only 19% reported living in a household with electricity, the highest proportion found at the facility in Bondo town (29%). Male and female children were equally brought to the facilities with an average child’s age of 31 months. Less than 2% of caregivers had not had any formal education; 93.5% had reached or completed primary or secondary while post-secondary education was more common among caregivers at Bondo district hospital (10%). Nearly all (97.3%) caregivers were able to read either in Kiswahili, Dholuo or English; more commonly in Kiswahili (93.8%) than in English (87.5%), while the ability to read vernacular Dholuo was less common at only one site (82%). On average caregivers reached facilities from their homes within half an hour with the large majority either walking or using low cost bicycles *“boda-boda”* depending on the site and the availability of these transport facilities (Table 1).

**Table 1:** General characteristics of caregivers, by study site

	**Bondo DH N = 100**	**Got Agulu SDH N = 100**	**Ndori HC N = 100**	**Madiany DH N = 100**	**All sites N = 400**
**Female**	94 (94%)	95 (95%)	94 (94%)	91 (91%)	374 (93.5%)
**Age (mean in years)**	28	25	28	27	27
**Brought female child**	53 (53%)	48 (48%)	50 (50%)	47 (47%)	198 (49.5%)
**Child’s age (mean in months)**	31.0	28.2	30.0	34.3	30.9
**Relation to child**					
Mother	91 (91%)	93 (93%)	81 (81%)	86 (86%)	351 (87.8%)
Father	6 (6%)	2 (2%)	3 (3%)	5 (5%)	16 (4.0%)
Grandmother	2 (2%)	1 (1%)	5 (5%)	4 (4%)	12 (3.0%)
Other^a^	1 (1%)	4 (4%)	11 (11%)	5 (5%)	21 (5.3%)
**Education**					
No formal education	1 (1%)	2 (2%)	3 (3%)	1 (1%)	7 (1.8%)
Primary	60 (60%)	68 (68%)	74 (74%)	66 (66%)	268 (67.0%)
Secondary	29 (29%)	26 (26%)	21 (21%)	30 (30%)	106 (26.5%)
Post-secondary	10 (10%)	4 (4%)	2 (2%)	3 (3%)	19 (4.8%)
**Able to read**					
Kiswahili	94 (94%)	91(91%)	97 (97%)	93 (93%)	375 (93.8%)
Dholuo	92 (92%)	82 (82%)	97 (97%)	96 (96%)	367 (91.8%)
English	86 (86%)	91 (91%)	81 (81%)	92 (92%)	350 (87.5%)
Other^b^	1 (1%)	18 (18%)	2 (2%)	2 (2%)	23 (5.8%)
**Reached facility by**					
Walking	42 (42%)	34 (34%)	86 (86%)	60 (60%)	222 (55.5%)
Boda boda	52 (52%)	65 (65%)	10 (10%)	40 (40%)	167 (41.8%)
Bus/matatu	6 (6%)	1 (1%)	4 (4%)	0	11 (2.8%)
**Has electricity at home**	29 (29%)	22 (22%)	17 (17%)	8 (8%)	76 (19.0%)

^a^includes aunt (13), sister (4), grandfather (2), brother (1) and uncle (1).

^b^includes Luhya (19), French (1), German (1), Kalenjin (1) and Kamba (1).

### Network, access, ownership and use of mobile phones

Mobile network signal at caregivers’ homes was nearly universal (99.8%) (Table 2). The large majority (93%; site range: 89-98%) of caregivers had access to mobile phone within the household. Nearly three-quarters (74%) owned personal phones (site range: 66-78%). Among 77 caregivers having access to someone else’s phone, 78% used their husband’s phone. Over three-quarters (77%) of phone owners had only one phone number while among the remaining 68 caregivers with two numbers 94% never change their SIM cards mainly due to use of dual card phones.

**Table 2:** Mobile network, access, ownership and use of phones, by study site

	**Bondo DH**	**Got Agulu SDH**	**Ndori HC**	**Madiany DH**	**All sites**
**Network coverage, phone access and ownership**	**N = 100**	**N = 100**	**N = 100**	**N = 100**	**N = 400**
Mobile network at home	100 (100%)	99 (99.0%)	100 (100%)	100 (100%)	399 (99.8%)
Has access to mobile phone	98 (98%)	90 (90%)	95 (95%)	89 (89%)	372 (93.0%)
Has personal mobile phone	78 (78%)	66 (66%)	73 (73%)	78 (78%)	295 (73.8%)
**Use of mobile phones**	**N = 98**	**N = 90**	**N = 95**	**N = 89**	**N = 372**
Voice	98 (100%)	90 (100%)	95 (100%)	89 (100%)	372 (100%)
Receive SMS	97 (99.0%)	86 (95.6%)	89 (93.7%)	76 (85.4%)	348 (93.6%)
Send SMS	91 (92.9%)	81 (90.0%)	83 (87.4%)	74 (83.2%)	329 (88.4%)
Money transfer	92 (93.9%)	79 (87.8%)	74 (77.9%)	81 (91.0%)	326 (87.6%)
Browsing	9 (9.2%)	13 (14.4%)	4 (4.2%)	19 (21.4%)	45 (12.1%)
E-mail	5 (5.1%)	12 (13.3%)	2 (2.1%)	17 (19.1%)	36 (9.7%)
**Brought phone to the facility**	63 (64.3%)	44 (48.9%)	49 (51.6%)	56 (62.9%)	212 (57.0%)
**Able to charge phone at home**	29 (29.6%)	22 (24.4%)	17 (17.9%)	8 (9.0%)	76 (20.4%)
**Patterns of SMS receiving**	**N = 97**	**N = 86**	**N = 89**	**N = 76**	**N = 348**
No of SMS/week (median[IQR])^a^	5 [3-10]	7 [5-10]	5 [3-10]	21 [14–35]	9 [4-20]
Able to open and read SMS	96 (99.0%)	86 (100%)	89 (100%)	76 (100%)	347 (99.7%)
Reading of SMS (day time)^a^					
Immediately	88 (90.7%)	75 (88.2%)	68 (76.4%)	72 (94.7%)	303 (87.3%)
Within 1 hour	9 (9.3%)	6 (7.1%)	20 (22.5%)	3 (4.0%)	38 (11.0%)
After 1 hour	0	4 (4.7%)	1 (1.1%)	1 (1.3%)	6 (1.7%)
Reading of SMS (night time)					
Immediately	23 (23.7%)	33 (38.8%)	75 (84.3%)	29 (38.2%)	160 (46.1%)
In the morning	74 (76.3%)	52 (61.2%)	14 (15.7%)	47 (61.8%)	187 (53.9%)

^a^Denominators for these variables at Got Agulu SDH and at all sites do not include one observation with missing value.

Mobile phones were brought to the facility by 212 (57%) of caregivers, more commonly by those having personal phones (71%) than those having access to someone else’s phone (5%). Two-thirds (65.4%) of caregivers had basic phones, one-third (32.7%) medium level ones while only 1.7% caregivers had “smartphones”. Only 3.3% of phones had empty battery at the time of the interview. Among phone owners the most common reason for not bringing phone to the facility was charging of phone (87.5%). Despite only 19% of caregivers having electricity at home and the majority (63%) charging phones at nearby shopping centres, nearly all (99.7%) reported that they would be able in the next 28 days to have permanent access to phones to receive text-messages.

Among caregivers having access to phones, all used the voice function of the phone to make or receive calls and a large majority (94%; site range: 85-99%) also reported using phones to receive text-messages (Table 2). The median number of text messages received per week was 9 and nearly all caregivers (99.7%) responded that they are able to open and read messages by themselves. The use of other phone functions such as those to send text-messages and do SMS money transfer were also widespread (88%), however, the use of phones for data transfer such as internet browsing and e-mail was less common (12% and 10% respectively). When a text-message was received during the day-time, nearly all caregivers (98.3%) reported that they read it either immediately or within one hour after its receipt. Interestingly, all caregivers reported receiving messages at night and 46% reported that they would read such messages immediately they arrived, even if they had been asleep.

### Willingness to receive text-message reminders and language preferences

The caregivers’ willingness to receive text message reminders was very high. All reported that they would like to receive text-message reminders about giving medicines to their child and nearly all (99.7%) expressed a wish to receive reminders to bring their child back for post-treatment review. Greater variability was observed with respect to language preferences. English was the text-messaging language of choice for 41.7% of respondents (site range: 26.3-51.7%), Kiswahili for 32.3% (site range: 16.9-46.7%) and Dholuo for 26.1% (site range: 14.4-34.7%).

## Discussion

The feasibility, patterns of use and acceptability of using mobile phone text-messaging to improve malaria treatment adherence and post-treatment review was investigated in an area of high malaria risk in Kenya. Despite some concerns that feasibility may be severely compromised by the rural setting; the target population of children and women; poor health and economic indicators; and limited education and literacy levels, we found highly favourable conditions for testing the impact of the proposed intervention. The findings have shown that the large majority of caregivers attending public facilities with children sick with malaria are able to read (97%); live in households covered by a mobile phone network (100%); have high mobile phone access (93%) and personal ownership (74%); and are commonly using the text-messaging phone function (94%). The impact evaluations of text-messaging interventions targeting caregivers of children with malaria are, therefore, not premature but indeed timely in this study setting.

The coverage with a mobile network will ultimately determine the feasibility of any mHealth intervention. The findings among caregivers’ attending public facilities within the study area are in line with the national reports of over 90% of signal coverage among similar populations [19]. They are however higher than in many African countries where the absence of a mobile signal may be the major impediment to effective mHealth interventions [23]. Importantly, very high mobile phone penetration and indeed significantly higher access and personal ownership was observed than found in household surveys among the general population in 2010 in Nyanza Province (access 57%, ownership 26%) [24], in 2011 among mothers of newborn children in one village in a neighbouring area (ownership 26%) [25], and nationally in 2012 in Kenya among caregivers presenting to public health facilities (access 86%, ownership 61%) [19]. Despite limitations of the comparisons due to different definitions, age groups and study populations, these optimistic 2013 results suggest continued growth in mobile phone penetration in Kenya providing feasibility reassurance for the study without the need for supply of mobile devices.

The characteristics of the study population, the patterns of mobile phone use and the willingness of caregivers to receive text-message reminders provided important reassurances and additional challenges to be addressed during the intervention development process. First, despite the fact that over 90% of caregivers’ are able to read the national language (Kiswahili), there was high variability in language preferences suggesting that the optimum intervention should provide an option of language selection among the three most commonly used languages in the area. Second, the finding that over 90% of caregivers already use mobile phones to receive text-messages with all of them able to open and read messages by themselves, suggests that caregivers’ orientation on the use of SMS function would be rarely required and consideration of an alternative delivery mode based on voice reminders is by in large redundant [26]. Third, the risk of non-exposure due to exchange of SIM cards is very low since over three-quarters of caregivers have only one phone number and those having two numbers mainly use dual card phones. Fourth, it was found that even when phones are owned, some caregivers may not bring the device with them to the facility. While mobile devices are not required for registration into the intervention and do not preclude the intervention delivery, attention will need to be paid to ensuring that correct phone numbers are communicated during the registration process. Fifth, charging of the phones outside of the households may be a major prohibiting barrier for timely access to the text-messages. Basic phones used by the large majority of caregivers which require short and uncommon charging, as well as caregivers’ responses that permanent access to text messages can be ensured at all times during the intervention delivery, provided some reassurance. However, attention will need to be paid to ensure that the wording of text-messages reflects possible transmission delays and that the evaluation of the intervention exposure includes SMS delivery reports or other appropriate methods of measuring timely exposure. Finally, despite the possibility of courtesy bias, the extent of nearly universal willingness of caregivers to receive text-message reminders suggests that SMS based interventions are timely and appealing to the population in study area.

## Conclusions

Despite concerns that the feasibility of mHealth text-messaging interventions targeting women and children may be severely compromised in rural, high malaria risk areas in Kenya, very favourable conditions were found with respect to mobile network, access and ownership of phones, use of text-messaging and minimum literacy levels required for successful intervention delivery. Moreover, there is a high willingness of caregivers attending public health facilities to receive text-message reminders for medication adherence and post-treatment review. The impact evaluations of carefully tailored text-messaging interventions targeting caregivers of children with malaria are timely and justified.

## Competing interests

All authors declared no competing interests.

## Authors’ contributions

All authors contributed to study design and development of questionnaires. GO, SG and DZ trained field workers. GO supervised the field work and analysed data. GO and DZ produced the first draft of the manuscript. All authors critically reviewed the paper and approved the final version.

## References

[B1] Earth Institute Barriers and Gaps Affecting mHealth in Low and Middle Income Countries: A Policy White Paper 2010 Washington, D.C: mHealth Alliance http://mhealthalliance.org/images/content/publications/barriers_and_gaps.pdf

[B2] WHO Global Observatory for eHealth New horizons for health through mobile technologies 2011 Geneva: World Health Organization http://www.who.int/goe/publications/goe_mhealth_web.pdf

[B3] Free C Phillips G Watson L Galli L Felix L Galli L Patel V Edwards P The effectiveness of mobile-health technologies to improve health care service delivery processes: a systematic review and meta-analysis PLoS Med 2013 10 e1001363 10.1371/journal.pmed.1001363 23458994 PMC3566926

[B4] Free C Phillips G Galli L Watson L Watson L Felix L Edwards P Patel V Haines A The effectiveness of mobile-health technology-based health behaviour change or disease management interventions for health care consumers: a systematic review PLoS Med 2013 10 e1001362 10.1371/journal.pmed.1001362 23349621 PMC3548655

[B5] Bastawrous A Armstrong MJ Mobile health use in low- and high-income countries: an overview of the peer-reviewed literature J R Soc Med 2013 106 130 142 10.1177/0141076812472620 23564897 PMC3618170

[B6] Tomlinson M Rotheram-Borus MJ Swartz L Tsai AC Scaling Up mHealth: Where is the evidence? PLoS Med 2013 10 e1001382 10.1371/journal.pmed.1001382 23424286 PMC3570540

[B7] Lester RT Ritvo P Mills EJ Kariri A Karanja S Chung MH Jack W Habyarimana J Sadatsafavi M Najafzadeh M Marra CA Estambale B Ngugi E Ball TB Thabane L Gelmon LJ Kimani J Ackers M Plummer FA Effects of a mobile phone short message service on antiretroviral treatment adherence in Kenya (WelTel Kenya1): a randomised trial Lancet 2010 376 1838 1845 10.1016/S0140-6736(10)61997-6 21071074

[B8] Pop-Eleches C Thirumurthy H Habyarimana JP Zivin JG Goldstein MP De Walque D MacKeen L Haberer J Kimaiyo S Sidle J Ngare D Bangsberg DR Mobile phone technologies improve adherence to antiretroviral treatment in a resource-limited setting: a randomized controlled trial of text message reminders AIDS 2011 25 825 834 10.1097/QAD.0b013e32834380c1 21252632 PMC3718389

[B9] Odeny TA Bailey RC Bukusi EA Simoni JM Tapia KA Yuhas K Holmes KK McClelland RS Text messaging to improve attendance at post-operative clinic visits after adult male circumcision for HIV prevention: a randomized controlled trial PloS One 2012 7 e43832 10.1371/journal.pone.0043832 22957034 PMC3434192

[B10] Lund S Hemed M Nielsen B Said A Said K Makungu M Rasch V Mobile phones as a health communication tool to improve skilled attendance at delivery in Zanzibar: a cluster-randomised controlled trial BJOG 2012 119 1256 1264 10.1111/j.1471-0528.2012.03413.x 22805598

[B11] Zurovac D Talisuna AO Snow RW Mobile phone text messaging: tool for malaria control in Africa PLoS Med 2012 9 e1001176 10.1371/journal.pmed.1001176 22363212 PMC3283546

[B12] Beer N Ali AS Rotllant G Abass AK Omari RS Al-mafazy AW Björkman A Källander K Adherence to artesunate-amodiaquine combination therapy for uncomplicated malaria in children in Zanzibar, Tanzania Trop Med Int Health 2009 14 1 9 19549001 10.1111/j.1365-3156.2009.02289.x

[B13] Gerstl S Dunkley S Mukhtar A Baker S Maikere J Successful introduction of artesunate combination therapy is not enough to fight malaria: results from an adherence study in Sierra Leone Trans R Soc Trop Med Hyg 2010 104 328 335 10.1016/j.trstmh.2009.12.008 20129636

[B14] Lawford H Zurovac D O’Reilly L Hoibak S Cowley A Munga S Vulule J Juma E Snow RW Allan R Adherence to prescribed artemisinin-based combination therapy in Garissa and Bunyala districts, Kenya Malar J 2011 10 281 10.1186/1475-2875-10-281 21943224 PMC3189920

[B15] Mace KE Mwandama D Jafali J Luka M Filler SJ Sande J Ali D Kachur SP Mathanga DP Skarbinski J Adherence to treatment with artemether-lumefantrine for uncomplicated malaria in rural Malawi Clin Infect Dis 2011 53 772 779 10.1093/cid/cir498 21921220

[B16] Onyango EO Ayodo G Watsierah CA Were T Okumu W Anyona SB Raballah E Okoth JM Gumo S Orinda GO Ouma C Factors associated with non-adherence to artemisinin-based combination therapy (ACT) to malaria in a rural population from holoendemic region of western Kenya BMC Infect Dis 2012 12 143 10.1186/1471-2334-12-143 22726955 PMC3482576

[B17] World Health Organization Global Plan for Artemisinin Resistance Containment 2011 Geneva: World Health Organization http://www.who.int/malaria/publications/atoz/9789241500838/en/index.html

[B18] Wesolowski A Eagle N Noor AM Snow RW Buckee CO Heterogeneous mobile phone ownership and usage patterns in Kenya PLoS One 2012 7 e35319 10.1371/journal.pone.0035319 22558140 PMC3338828

[B19] Zurovac D Otieno G Kigen S Mbithi A Muturi A Snow RW Nyandigisi A Ownership and use of mobile phones among health workers, caregivers of sick children and adult patients in Kenya: cross-sectional national survey Global Health 2013 9 20 10.1186/1744-8603-9-20 23672301 PMC3695884

[B20] Kenya National Bureau of Statistics and ICF Macro Kenya Demographic and Health Survey 2008–2009 2010 Calverton, Maryland: Kenya National Bureau of Statistics and ICF Macro

[B21] Hamel M Adazu K Obuor D Sewe M Vulule J Williamson JM Slutsker L Feikin DR Laserson KF Reversal in reductions in child mortality in Western Kenya, 2003–2009 Am J Trop Med Hyg 2011 85 597 605 10.4269/ajtmh.2011.10-0678 21976557 PMC3183762

[B22] Noor AM Kinyoki DK Ochieng JO Kabaria CW Alegana VA Otieno VA Kiptui R Soti D Yé Y Amin AA Snow RW The epidemiology and control profile of malaria in Kenya: reviewing the evidence to guide the future vector control 2012 Oxford: Division of Malaria Control, Ministry of Public Health and Sanitation & Malaria Public Health Department, KEMRI-Welcome Trust-University of Oxford Research Programme

[B23] Deloitte, GSMA Sub-Saharan Africa Mobile Observatory 2012 http://www.gsma.com/publicpolicy/wp-content/uploads/2012/03/SSA_FullReport_v6.1_clean.pdf

[B24] Kenya National Bureau of Statistics National ICT Survey Report 2010 http://www.cck.go.ke/resc/downloads/REPORT_OF_THE_NATIONAL_ICT_SURVEY_2010.pdf

[B25] Wakadha H Chandir S Were EV Rubin A Obor D Levine OS Gibson DG Odhiambo F Laserson KF Feikin DR The feasibility of using mobile-phone based SMS reminders and conditional cash transfers to improve timely immunization in rural Kenya Vaccine 2013 31 987 993 10.1016/j.vaccine.2012.11.093 23246258 PMC4603391

[B26] De Costa A Shet A Kumarasamy N Ashorn P Eriksson B Bogg L Diwan VK Design of a randomized trial to evaluate the influence of mobile phone reminders on adherence to first line antiretroviral treatment in South India–the HIVIND study protocol BMC Med Res Methodol 2010 10 25 10.1186/1471-2288-10-25 20346136 PMC2858730

